# Characterizing and Minimizing Aggregation and Particle Formation of Three Recombinant Fusion-Protein Bulk Antigens for Use in a Candidate Trivalent Rotavirus Vaccine

**DOI:** 10.1016/j.xphs.2019.08.001

**Published:** 2020-01-01

**Authors:** Sanjeev Agarwal, Neha Sahni, John M. Hickey, George A. Robertson, Robert Sitrin, Stanley Cryz, Sangeeta B. Joshi, David B. Volkin

**Affiliations:** 1 Department of Pharmaceutical Chemistry, Vaccine Analytics and Formulation Center, University of Kansas, 2030 Becker Drive, Lawrence, Kansas 66047; 2 The Center for Vaccine Innovation and Access, PATH, 455 Massachusetts Avenue NW Suite 1000, Washington, District of Columbia 20001

**Keywords:** formulation, recombinant protein, subunit vaccine, rotavirus, stability, aggregation

## Abstract

In a companion paper, the structural integrity, conformational stability, and degradation mechanisms of 3 recombinant fusion-protein antigens comprising a non-replicating rotavirus (NRRV) vaccine candidate (currently being evaluated in early-stage clinical trials) are described. In this work, we focus on the aggregation propensity of the 3 NRRV antigens coupled to formulation development studies to identify common frozen bulk candidate formulations. The P2-VP8-P[8] antigen was most susceptible to shaking and freeze-thaw–induced aggregation and particle formation. Each NRRV antigen formed aggregates with structurally altered protein (with exposed apolar regions and intermolecular β-sheet) and dimers containing a non-native disulfide bond. From excipient screening studies with P2-VP8-P[8], sugars or polyols (e.g., sucrose, trehalose, mannitol, sorbitol) and various detergents (e.g., Pluronic F-68, polysorbate 20 and 80, PEG-3350) were identified as stabilizers against aggregation. By combining promising additives, candidate bulk formulations were optimized to not only minimize agitation-induced aggregation, but also particle formation due to freeze-thaw stress of P2-VP8-P[8] antigen. Owing to limited material availability, stabilization of the P2-VP8-P[4] and P2-VP8-P[6] was confirmed with the lead candidate P2-VP8-P[8] formulations. The optimization of these bulk NRRV candidate formulations is discussed in the context of subsequent drug product formulations in the presence of aluminum adjuvants.

## Introduction

Essentially every child before reaching 5 years of age gets infected by Rotavirus (RV), which can cause gastroenteritis and diarrhea.^1^ There are currently 4 WHO prequalified RV vaccines (RotaTeq®, Rotarix®, Rotavac®, and Rotasil®) which combined cover over 100 countries to reduce the burden of this viral infection.^2^ In addition, there are several other live, attenuated oral RV vaccines approved for local use and approximately 5 more candidates are in clinical trials.^3^ Vaccine efficacy of live, orally administered RV vaccines varies considerably, however, between developing (~40%-60%) versus developed countries (~80%-90%).^4-7^ Thus, there is growing interest in a recombinant protein subunit vaccine with parenteral administration capabilities to address these differences and provide similar efficacy irrespective of the socio-economic background of a child.^8,9^ Success or failure of a vaccine also relies on its global coverage, and unfortunately, global coverage of rotavirus vaccines is currently only about 28%.^10^ Development of a recombinant subunit RV vaccine will hopefully also improve affordability, allow for a more a constant vaccine supply, and improve coverage by addition to widely used pediatric combination vaccines. For example, a subunit RV vaccine could eventually be combined with the current childhood combination vaccines such as hexavalent DTaP-IPV-HepB-Hib or the pentavalent DTwcP-HepB-Hib vaccines to improve compliance with the immunization schedule and encourage wider vaccination coverage.^8^

Although vaccine effectiveness is mainly guided by its composition (e.g., antigen and adjuvant), development of a stable vaccine formulation is equally important to ensure the safety and efficacy during manufacturing, long-term storage, transport, and administration.^11,12^ Live, attenuated viral vaccines contain weakened versions of the pathogens and are often sensitive to elevated temperatures and thus vulnerable to potency loss due to cold chain break down, especially in the developing world.^13^ By contrast, recombinant protein subunit vaccines are in general considered safer and more stable (although often require adjuvants to enhance immune responses).^14^ Considerable efforts have been made toward development of subunit RV vaccine candidates (e.g., soluble antigens, virus-like particles) that are being evaluated in preclinical and early clinical studies.^15-19^ One such candidate (referred to non-replicating rotavirus [NRRV]) containing a trivalent mixture of recombinant truncated VP8* fusion proteins is in phase 1/2 clinical trials. Previously, one of the 3 antigens (P2-VP8-P[8]) was shown to be safe in healthy adults as well as to be well tolerated and immunogenic in infants and toddlers thus establishing the proof of concept.^20,21^ See companion paper for detailed structural composition of the 3 NRRV antigens.^22^ Briefly, each antigen is composed of a universal tetanus toxoid CD4^+^ T cell epitope, P2, fused with a truncated DVP8* protein using a GSGSS linker. DVP8* is a soluble truncated version of the VP8* protein which is a proteolytically cleaved product of RV surface protein VP4. The 3 NRRV antigens are produced recombinantly in Escherichia coli as fusion proteins and are named as P2-VP8-P[4], P2-VP8-P[6], and P2-VP8-P[8] where P2 refers to the tetanus toxoid epitope and VP8-P[x] represents the DVP8* protein derived from human RV strain DS-1 (G2P[4]), 1076 (G2P[6]), or Wa (G1P[8]).^23,24^ The 3 antigens are abbreviated as P [4], P[6], and P[8], respectively, in this article.

For successful formulation development of a new recombinant protein antigen, the following steps including (1) analytical characterization of key structural attributes, (2) understanding of the physicochemical degradation mechanisms, and (3) rational design of formulation composition to minimize degradation are performed to maintain a vaccine antigen’s potency during storage over the intended period of use.^13^ Because protein molecules are only marginally stable in their native folded conformation, it is necessary to identify optimal solution conditions (e.g., buffer, pH, ionic strength, excipients, etc.) to ensure their integrity and stability at every step of the manufacturing process as well as during long-term storage.^25,26^ The vaccine formulation development effort becomes even more challenging when multiple protein antigens are combined into a multivalent vaccine candidate.^13,27^ During the course of manufacturing protein antigens for multivalent vaccines, it is convenient and often necessary for the manufacturer to separate the manufacturing of the purified antigen (bulk drug substance) from subsequent formulation and fill-finish operations (vaccine drug product). This is very useful and often necessary in a more limited manufacturing environment (such as in some developing countries) when the production and purification are carried out in a setting which can handle only one product at a time. In particular for multivalent vaccines it is therefore advantageous to campaign each antigen and store the purified drug substance, often without adjuvant in a frozen state.

The work described herein is a case study to characterize and minimize aggregation and particle formation, and to identify candidate stable frozen liquid formulations for bulk storage, of the 3 NRRV antigens for use in a trivalent subunit RV vaccine in the developing world. In a companion paper, physicochemical characterization and forced degradation studies of the 3 protein antigens are described to compare their structural integrity and physico-chemical stability profiles, and to develop analytical tools to monitor or quantify degradation products.^22^ The aggregation propensity (i.e., colloidal instability) of the antigens was identified as a major degradation mechanism. In addition, aggregation during handling and thawing of the frozen bulk drug substance at large scale has been observed (data not shown). Thus, not only was a better understanding of the conditions leading to physical instability of the 3 protein antigens pursued, but candidate frozen liquid formulations were proposed and evaluated to minimize such physical instability. Because the NRRV antigens are already in clinical trials, the selection of final formulation components was constrained such that minimal changes to the current formulation are preferred. In addition, the new candidate bulk formulations must be compatible with subsequent formulation steps with aluminum adjuvants.

## Materials and Methods

The P[4] and P[6] used for colloidal stressed stability studies were produced and purified from E. coli at Walter Reed Army Institute of Research, MD, and formulated in 0.5 mM sodium phosphate, 150 mM NaCl, pH 7.2. The P[4] and P[6] used for the bulk formulation studies, and P[8] used for all the studies in this work, were produced and purified from E. coli by Blue Sky BioServices, MA, and provided in 600 mM ammonium sulfate (AS), 50 mM Tris buffer at pH 7.5. Sodium phosphate dibasic heptahydrate and sodium chloride were purchased from Thermo Fisher Scientific (Waltham, MA). All other buffer reagents and chemicals including sodium phosphate monobasic monohydrate, citric acid, and ammonium bicarbonate were purchased from Sigma-Aldrich (St. Louis, MO) and were of analytical grade or higher unless noted otherwise. Protein concentration for each antigen was determined using extinction coefficient as described in the companion paper by Agarwal et al.^22^

Experimental details including sample preparation for colloidal stability studies and aggregate and particle characterization, excipient screening, and optimization studies including pH and buffer optimization, freeze-thaw (FT) studies are provided in the Supplemental Methods section. A “base buffer” of 10 mM sodium phosphate, 150 mM NaCl, pH 7.2 was chosen for initial characterization and excipient screening based on our prior work with these antigens (see companion paper). In addition, details of the methods used have mostly been described previously^28-30^ are provided in the Supplemental Methods section including visual appearance, turbidimetry, micro-flow imaging (MFI), UV-visible spectroscopy, resonant mass measurement, sedimentation velocity analytical ultracentrifugation (SV-AUC), size exclusion chromatography (SEC), SDS-PAGE, extrinsic 1-anilinonaphthalene-8-sulfonate (ANS) fluorescence spectroscopy, Fourier transform infrared (FTIR) spectroscopy and FTIR microscopy.

## Results

### Colloidal Stability Assessments and Characterization of Aggregates and Particles

**Figure 1:**
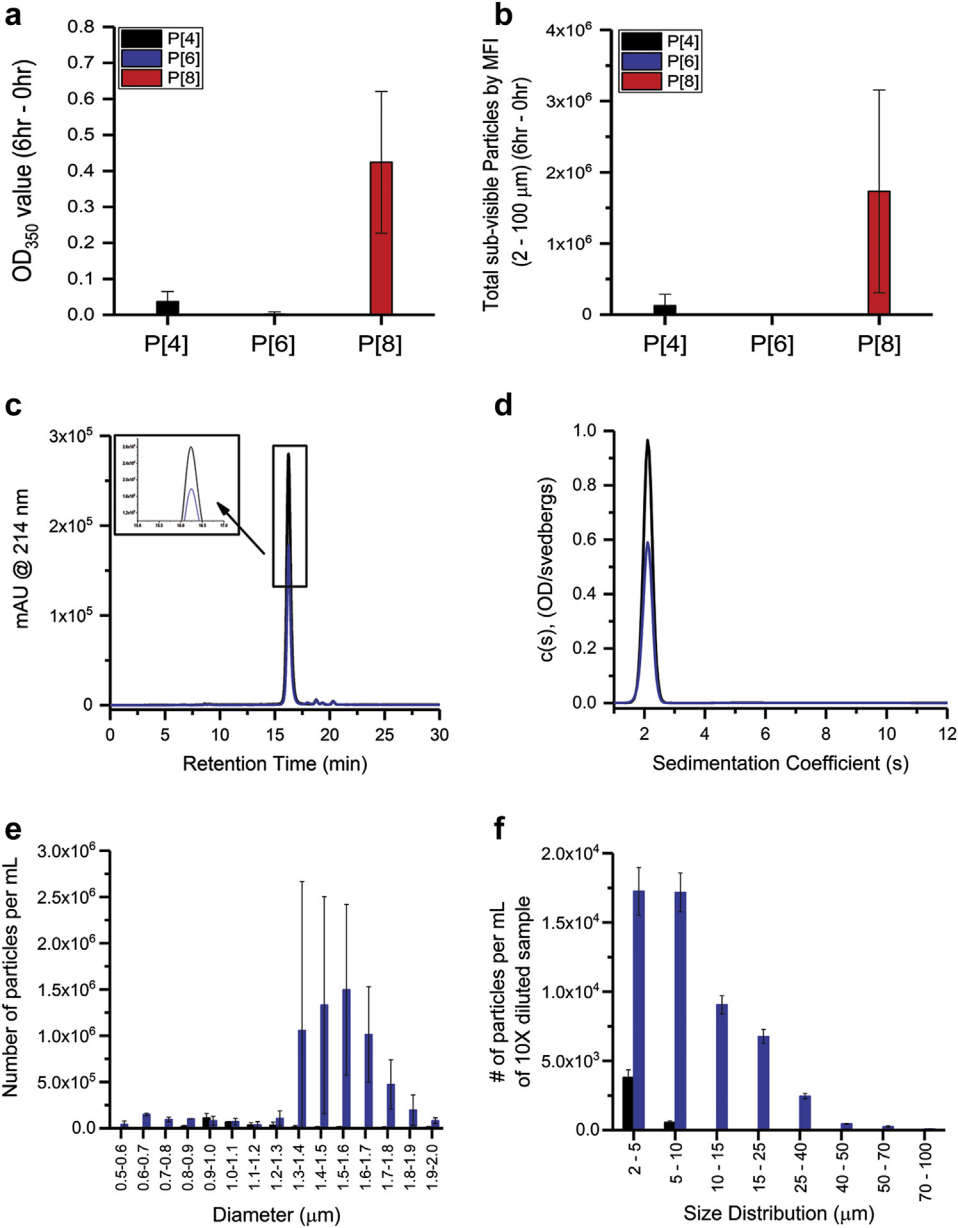
Colloidal stability assessment and comparison of the 3 NRRV antigens after shake stressed for 6 h. (a) Light-scattering as measured by OD350 values, and (b) total subvisible particles (2-100 μm) per mL by MFI. Results from unstressed samples (0 h) are subtracted from stressed samples (6 h). Size-distribution analysis of shake stressed P[8] antigen in base buffer (10 mM PBS pH 7.2) were then monitored as soluble aggregates/fragments and protein-loss quantification by (c) SEC, and (d) SV-AUC analysis. Submicron and subvisible particle analysis by (e) resonant mass measurement, and (f) micro–flow imaging microscopy, respectively. Error bars represent 1 SD from 3 separate measurements. Refer Table 1 for comparative data of similar shake studies for 1.5 h with P[4], P[6], and P[8] antigens.

The focus of this study is to better understand the nature and extent of aggregation and particle formation of the 3 NRRV antigens as well as to propose and evaluate new candidate bulk formulations to minimize such degradation. As an initial step, the 3 recombinant fusion-protein antigens were subjected to shaking or agitation stress and as shown in Figure 1a, high OD^350^ value of ~0.45 was observed for P[8] antigen which indicates slightly elevated protein aggregation compared to P[4] and P[6]. The P[6] protein appeared to be less susceptible to aggregation when subjected to shaking stress under the tested conditions (with OD^350^ value below 0.01). Because all 3 antigens showed similar turbidity values after shake stress (7.8-12.9 NTU; see Table 1), this result is likely a reflection of the formation of larger aggregates and particulates. Consistent with this, the total number of subvisible particles in the stressed samples was highest for P[8] and lowest for P[6] antigen as measured by micro-flow imaging (MFI) (Fig. 1b). As shown in Table 1, visible particles were observed by visual assessment in some of the stressed samples, and A^280^ values after centrifugation and light-scattering correction decreased by ~30% for P[4] and P[8], and ~35% for P[6] antigen indicating notable loss in protein mass. No detectable soluble aggregates were recorded by SEC and SV-AUC for each antigen; however, substantial monomer loss was observed (consistent with UV-visible spectroscopy results), suggesting the formation of larger, insoluble aggregates (Table 1). Figures 1c-1f show representative aggregate and particle size distribution data for P[8] antigen; no soluble aggregates were detected and only a major peak corresponding to monomer was observed by both SEC chromatograms and SV-AUC c(s) distribution analysis (Figs. 1c and 1d). Substantial increases in the larger subvisible particles in size ranges 1.3-1.8 μm and 2-40 μm were observed, however, in stressed P[8] samples by resonant mass measurement (Fig. 1e) and MFI (Fig. 1f) measurements, respectively.

**Table 1:** Comparison of Aggregate and Particle Formation in Different Size Ranges for the Three NRRV Antigens

Size Range	Analytical Methods		P[4]		P[6]		P[8]	
			T = 0 h	T = 1.5 h	T = 0 h	T = 1.5 h	T = 0 h	T = 1.5 h
>100 F020µm	Visual assessment of visible particles		–	–	–	+	+	+
1 nm-100 µm	UV-visible absorption spectroscopy	A280	0.35 ± 0.0	0.25 ± 0.01	0.35 ± 0.0	0.23 ± 0.0	0.37 ± 0.0	0.26 ± 0.0
		OD350	0.01 ± 0.0	0.46 ± 0.07	0.01 ± 0.0	0.35 ± 0.02	0.01 ± 0.0	0.40 ± 0.04
	Turbidimetry (NTU)		0.5 ± 0.0	12.9 ± 0.8	0.5 ± 0.0	7.8 ± 0.5	0.5 ± 0.0	9.9 ± 0.3
2-100 µm	Micro-flow imaging (total particles/mL of 10× diluted sample)		1.4 ± 0.4 × 10^3^	1.0 ± 0.2 × 10^5^	3.3 ± 0.5 × 10^3^	0.8 ± 0.1 × 10^5^	4.4 ± 0.7 × 10^3^	0.5 ± 0.0 × 10^5^
0.1-2 µm	Resonant mass measurement (total Particles/mL)		2.1 ± 0.1 × 10^5^	1.2 ± 1.0 × 10^6^	1.0 ± 0.1 × 10^6^	3.3 ± 2.2 × 10^7^	3.2 ± 1.0 × 10^5^	6.3 ± 3.9 × 10^6^
1-100 nm	SV-AUC	Monomer (%)	100 ± <1	70 ± 2	100 ± <1	74 ± 1	100 ± <1	67 ± 2
		Soluble aggregates (%)	0 ± <1	0 ± <1	0 ± <1	0 ± <1	0 ± <1	0 ± <1
	SEC	Monomer (%)	99.4 ± 0.1	71.4 ± 4.2	98.5 ± 0.3	74.7 ± 0.8	96.7 ± 0.1	65.2 ± 3.6
		Fragments (%)	0.3 ± 0.0	1.3 ± 0.4	0.2 ± 0.2	2.6 ± 0.4	3.3 ± 0.1	3.9 ± 0.1
		Soluble + insoluble aggregates (%)	0.3 ± 0.0	27.4 ± 4.3	1.3 ± 0.1	21.9 ± 1.2	0.0 ± 0.0	30.8 ± 3.8

Similar levels of particles were generated in each NRRV antigen under forced degradation conditions of shaking stress for 1.5 h at 250 RPM at room temperature in 10 Mm sodium phosphate 150 mM NaCl pH 7.2 buffer. Error bars represent 1 SD from triplicate measurements.

**Figure 2:**
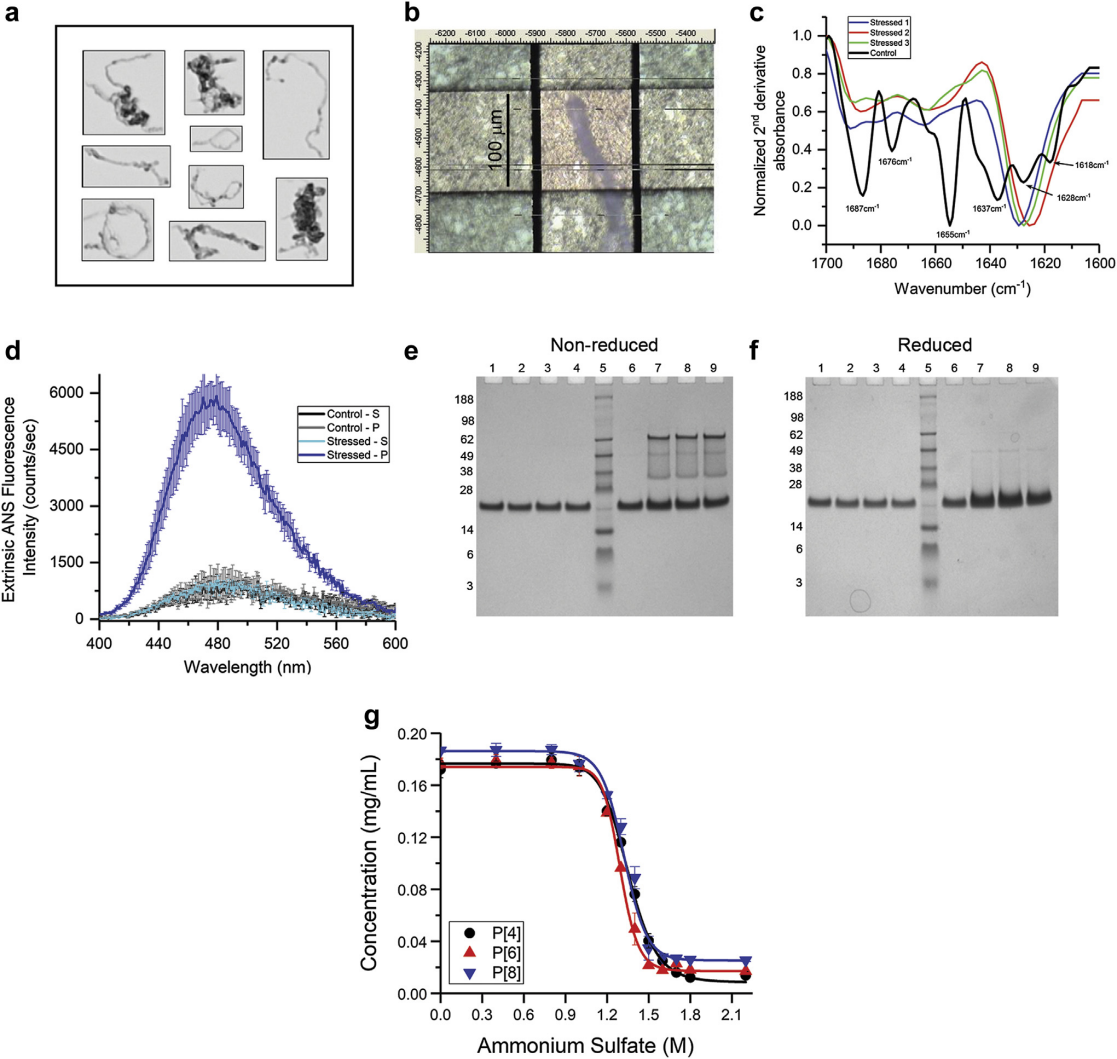
Morphology and structural analysis of protein within aggregates/particles formed for P[8] antigen after shake stress in base buffer. (a) Representative particle images recorded by micro–flow imaging microscopy, (b) optical microscopic image of an isolated protein particle/aggregate, (c) secondary structure analysis of protein in isolated insoluble protein particles/aggregates by FTIR microscopy, (d) higher order structure integrity analysis of unstressed and stressed protein in the supernatant (S) and pellet (P) fractions by ANS fluorescence spectroscopy, and inter-molecular disulfide bond analysis of unstressed and stressed protein in S and P fractions by (e) nonreduced and (f) reduced SDS-PAGE (lane 1—S unstressed, lanes 2,3,4—S stressed, lane 5—MW marker, lane 6—P unstressed, and lanes 7,8,9—P stressed). (g) Relative solubility assessment of 3 NRRV antigens using ammonium sulfate precipitation assay, error bars represent 1SD from triplicate experiments.

**Figure 3:**
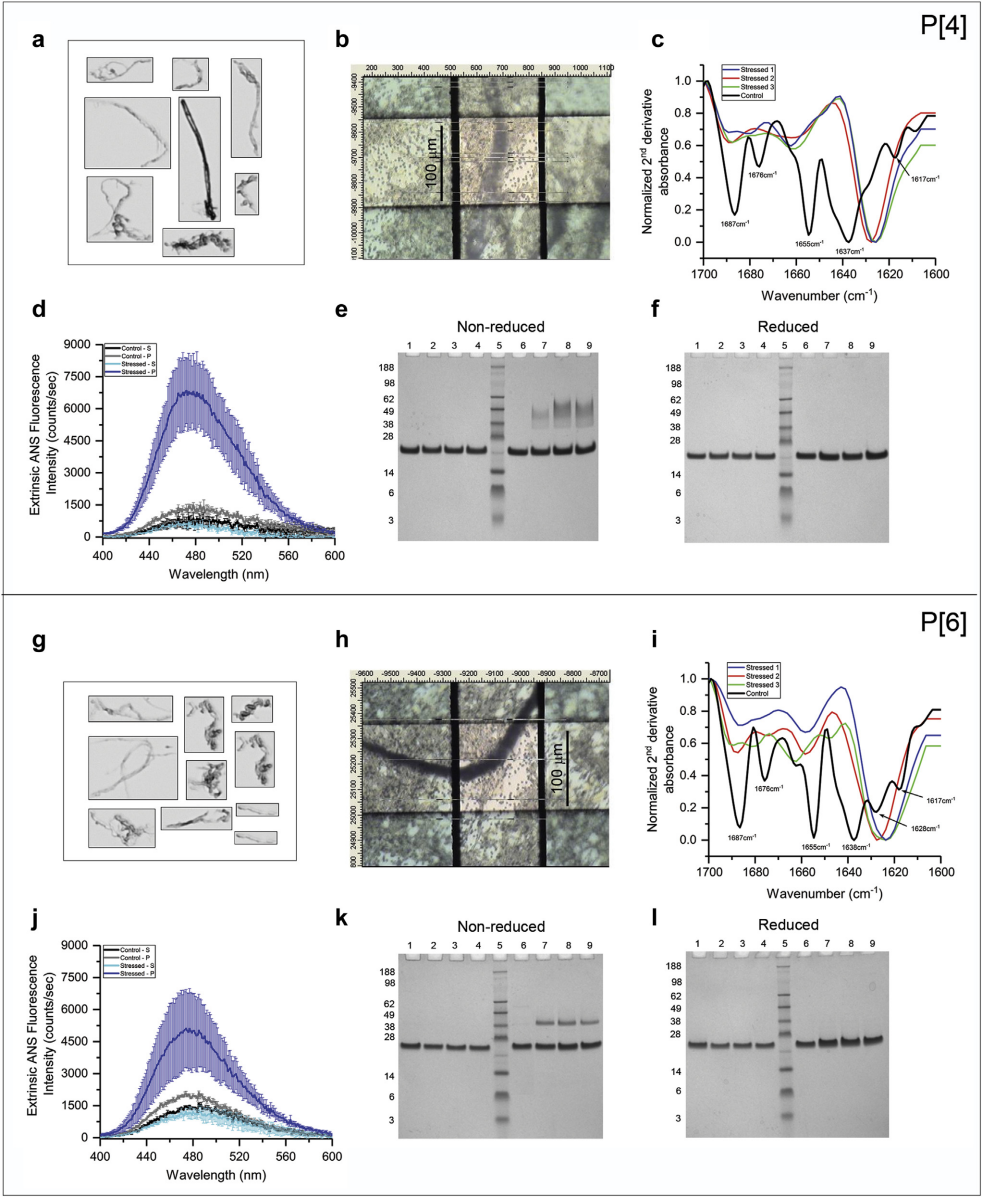
Morphology, higher-order structure and chemical composition of aggregates/particles formed for P[4] (top panel) and P[6] (bottom panel) antigens after shake stress in base formulation. (a and g) Representative particle images recorded by micro–flow imaging microscopy, (b and h) representative optical microscopic image of an isolated protein particle, (c and i) secondary structure analysis of unstressed protein in solution (control) and isolated particle from stressed sample by FTIR microscopy, (d and j) higher-order structure integrity analysis of unstressed and stressed protein in the supernatant (S) and pellet (P) fractions by ANS fluorescence spectroscopy. Inter-molecular disulfide bond analysis of unstressed and stressed protein in S and P fractions by (e and k) nonreduced and (f and l) reduced SDS-PAGE (lane 1—S unstressed, lanes 2,3,4—S stressed, lane 5—MW marker, lane 6—P unstressed, and lanes 7,8,9—P stressed).

The aggregates and particles generated from shake stress were then further evaluated in terms of morphology, higher-order structure (FTIR analysis and hydrophobic exposure by ANS fluorescence), and chemical composition (non-native disulfide formation). The results for P[8] antigen are described in Figure 2 and for P[4] and P[6] antigens in Figure 3. First, analysis of the MFI images revealed that the micron size particles formed were opaque and fibrillar in morphology for each antigen (Figs. 2a, 3a, and 3g). The optical microscope was used to visualize the isolated particle (Figs. 2b, 3b, and 3h). FTIR analysis was then utilized to examine the overall secondary structure content of the unstressed sample in solution and FTIR microscopic analysis was used to examine the secondary structure of protein within the isolated particles generated by shake-stress. Second derivative of amide I FTIR spectra were notably different for isolated P[8] particles versus control protein in solution (Figs. 2c, 3c, and 3i). Many of the peaks in the control sample FTIR spectra were not retained in the stressed sample spectra suggesting loss of native structure. In addition, an additional primary peak (~1625 cm^–1^) for protein within the isolated particles indicated the formation of intermolecular b-sheet sheets (i.e., aggregates) for each stressed antigen sample. Alterations in higher-order structural integrity of the NRRV protein present in the isolated particles (vs. control protein in solution) were assessed by measuring the fluorescence of ANS dye binding. As shown in Figures 2d, 3d, and 3j, substantial increase in the fluorescence intensity of ANS was recorded for the pellet of the agitation stressed samples as compared to the supernatant of stressed samples as well as compared to the supernatant or pellet of control protein samples. The higher fluorescence intensity of the protein derived from the pellet suggested increased hydrophobic surfaces because of structural alterations or aggregate formation, or both due to shaking stress. SDS-PAGE analysis of the protein from the pellet generated after shaking indicated the presence of oligomeric species ranging from 38 to 68 kDa in addition to the monomeric protein under nonreducing conditions (Figs. 2e, 3e, and 3k). The oligomeric species were not present in the reducing gel (Figs. 2f, 3f, and 3l) suggesting they were linked through non-native intermolecular disulfide bonds (each NRRV antigen has single Cys residue). Single monomeric band was observed by SDS-PAGE for the control samples and for supernatant of the stressed samples under nonreduced and reduced conditions. Overall, the particles or aggregates generated for each NRRV antigen were opaque and fibrillar in morphology, showed increased intermolecular β-sheet content and hydrophobic exposure, and were partially crossed-linked with intermolecular disulfide bonds.

To assess if these agitation stress results, in terms of relative aggregate and particle formation of each antigen, are correlated with their relative solubility, the relative apparent solubility of each antigen was determined using an AS precipitation assay. As shown in Figure 2g, decreases in protein concentration were observed with the increasing amounts of AS added to the solution presumably due to the known salting-out mechanism of kosmotropic salts. The AS^midpt^ value was calculated for each antigen which is a measure of relative solubility by comparing the amount of AS needed to precipitate 50% of the protein out of solution. A similar AS^midpt^ value of 1.35 ± 0.01 M was observed for P[4] and P[8], whereas AS^midpt^ was 1.29 ± 0.01 M for P[6] suggesting an overall similar, albeit somewhat lower relative solubility of P[6]. This result is consistent with the relatively more hydrophobic nature of P[6] compared to the other 2 NRRV antigens (refer to companion paper by Agarwal et al.^22^). The more notable agitation induced aggregate and particle formation of the P[8] antigen is thus not consistent with rank ordering of relative apparent solubility (compared to the P[4] and P [6] antigens as measured by AS^midpt^ values), and is likely therefore due to other causes (e.g., differences in colloidal or interfacial properties; see Discussion).

### Evaluating Stabilizing Excipients to Minimize Shaking-Induced Aggregation of NRRV Antigens

**Figure 4:**
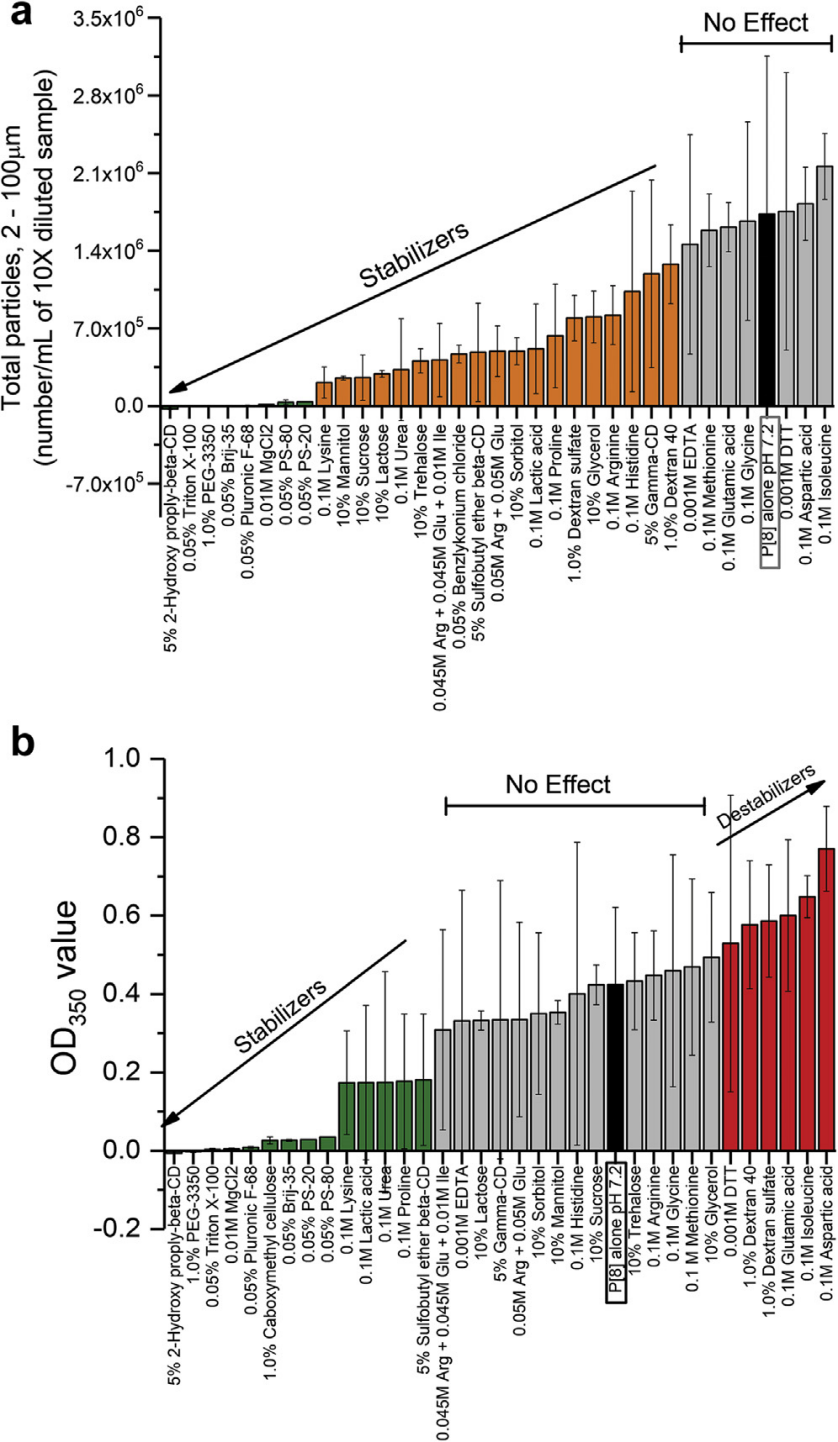
Excipient screening against agitation stress of P[8] protein antigen. (a) Total subvisible particles, and (b) OD350 values of 0.15 mg/mL P[8] solution after shake stressed for 6 h in base buffer (10 mM PBS pH 7.2; black bar, highlighted in box) and in base buffer containing different excipients. Excipients are rank-ordered from lowest to highest total subvisible particles or OD350 value suggesting highest to lowest stability. Excipients in green, orange, gray, and red resulted in large increase, moderate increase, no effect, and decrease in stability, respectively. Error bars represent 1 SD from triplicate experiments. Refer to Supplemental Figure S1 for similar studies with down-selected excipients for P[4] and P[6] antigens.

Owing to the susceptibility of NRRV antigens toward aggregation by shaking stress (see aforementioned) 35 pharmaceutical excipients were screened with P[8] antigen under stress to identify potential stabilizing excipients. Figure 4a shows total subvisible particles by MFI analysis (2-100 μm after 6 h of shaking minus time zero results per milliliter of 10× diluted sample) for P[8] in base buffer ± excipients. Many of the tested excipients mitigated particle formation during shaking stress (to varying extents), while the remaining excipients had no effect or were perhaps mildly destabilizing (Fig. 4a). Figure 4b shows the OD^350^ values (6 h-0 h) in an increasing order obtained from the spectra of P[8] antigen in the base buffer ± excipients. Overall, both the MFI and OD^350^ methods showed that many detergents (e.g., Triton X-100, Pluronic F-68, Brij-35, PS-20, and PS-80) were able to mitigate shaking induced aggregation of P[8] antigen. In addition, 2-OH propyl β-CD, PEG-3500, and MgCl^2^ also showed stabilizing effects. Owing to limited availability of P[4] and P[6] antigens, a subset of excipients that showed stabilizing effect on P[8] were tested with P[4] and P[6] as shown in Sup. Figure S1. Of this subset, each of the tested excipients had stabilizing effect on P[4] antigen, and with 2-OH propyl b-CD and PS-80 having the most positive effect (Sup. Figs. S1a and S1b). With P[6] antigen, the goal was to probe the compatibility of these excipients and look for significant detrimental effects since P[6] antigen (without any excipient) showed minimal aggregation under the tested shaking stress condition. As shown in Sup. Figures S1c and S1d, addition of the 6 excipients did not show any dramatic destabilization of P[6] antigen. Thus, 2-OH propyl b-CD, Pluronic F-68, and PS-80 were chosen for further optimization of their concentration with P[8] antigen under shaking stress.

**Figure 5:**
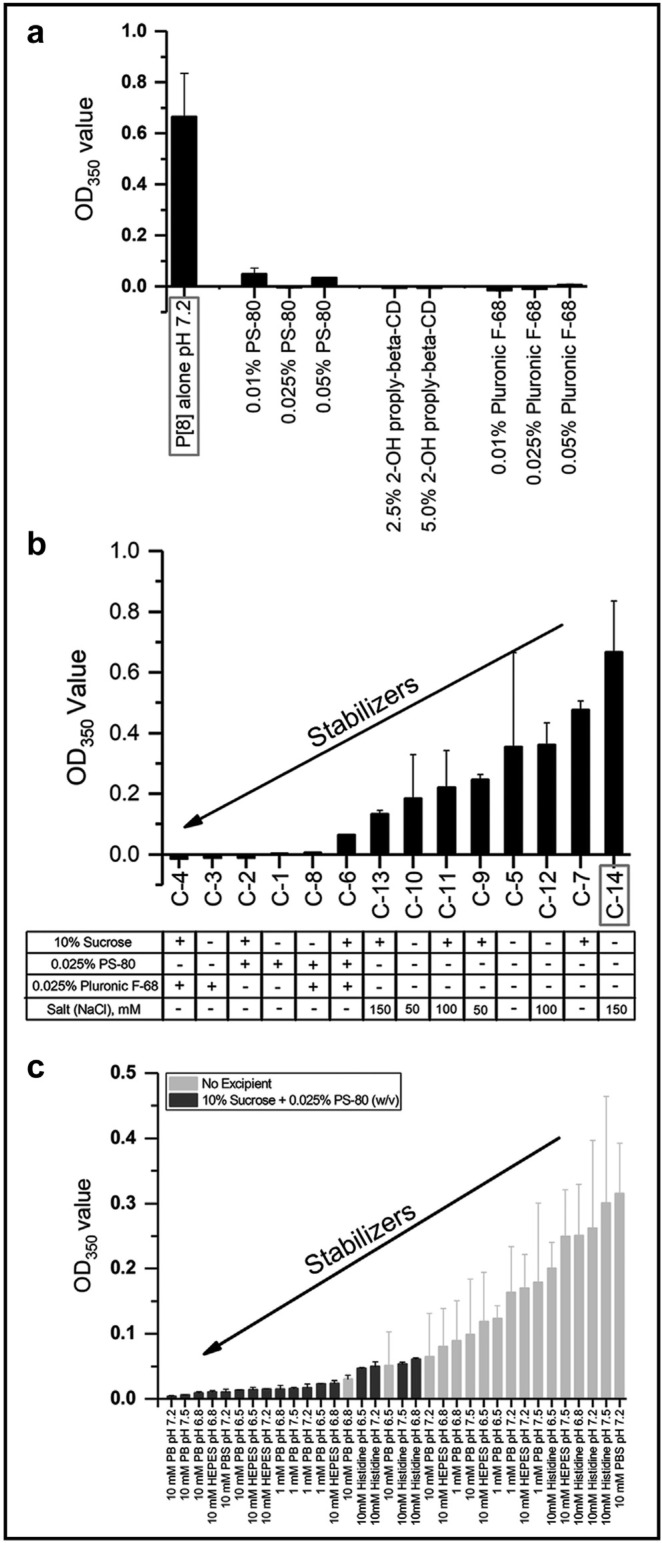
Effect of excipient concentrations and combinations as well as different buffer types and pH conditions on shaking induced aggregation propensity of P[8]. OD350 value of P[8] samples stressed for 6 h minus time zero samples with different (a) excipients concentrations, (b) excipients combination, and (c) different buffer types and pH conditions. Error bars represent 1 SD from triplicate experiments.

The concentration of each of the lead excipients was titrated down from the concentration used in the screening study to find the minimal effective concentration. As shown in Figure 5a, each of the selected excipients at lower concentrations was also able to mitigate protein aggregation. PS-80 was found to be most effective at 0.025% level out of the 3 tested concentrations. For 2-OH propyl b-CD and Pluronic F-68, all concentrations examined were equally effective. As the next step, different combinations of 0.025% (w/v) PS-80, 0.025% (w/v) Pluronic F-68, and 10% (w/v) sucrose (which minimized thermally induced aggregation, see below) with varying amounts of NaCl (0-150 mM) were tested for their synergistic effect on mitigating aggregation and particle formation due to shaking stress. As shown in Figure 5b, all the tested combinations out-performed the base formulation (C-14 in the figure which shows the highest P[8] aggregation propensity). Addition of 0.025% nonionic detergent either alone or in combination with 10% sucrose were able to mitigate aggregation (Fig. 5b; C-1-C-4, C8). Interestingly, detectable aggregates were observed in the stressed P[8] samples containing all 3 excipients (C-6). As expected, sucrose alone or in combination with different amounts of salt (C-13, C-11, C-9) was not able to minimize shaking-induced aggregation of P[8]. Furthermore, 3 different buffering agents (sodium phosphate, Histidine, and HEPES) were evaluated at 4 pH values (6.5, 6.8, 7.2, and 7.5) for their effect(s) with the P[8] antigen in the C-2 formulation (10% Sucrose, 0.025% PS-80) as shown in Figure 5c. Phosphate and HEPES buffers had similar effects and were better than histidine (10 mM phosphate was marginally better than 1 mM phosphate buffer) and no apparent effect of pH was observed on shaking-induced aggregation of P[8].

**Figure 6:**
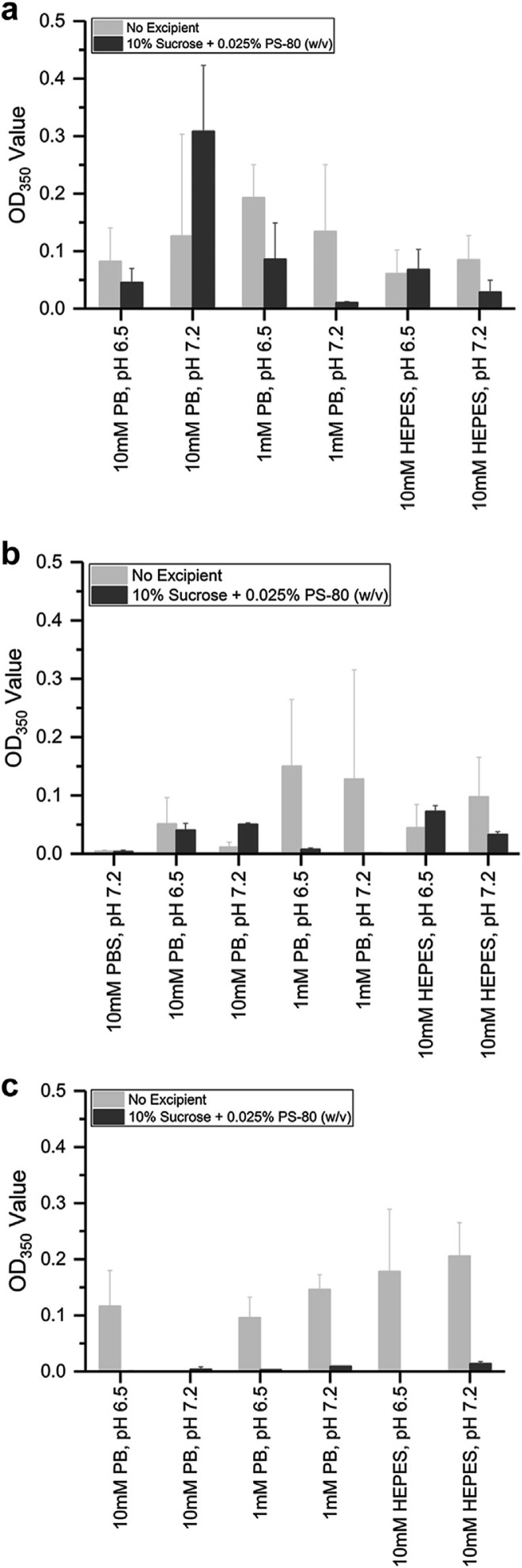
Effects of top excipient combinations and solution conditions on shaking induced aggregation propensity of NRRV antigens as measured by OD350 analysis. (a) P[4] antigen, and (b) P[6] antigen with and without indicated excipients. (c) Shaking-induced aggregation propensity of P[4] in the presence of 150 mM NaCl with and without indicated excipients. Error bars represent 1 SD from triplicate experiments.

Based on the results described previously with P[8] antigen, excipient combination, pH conditions, and buffer systems were down-selected to test with P[4] and P[6] antigens (due to their limited availability). As shown in Figure 6a, surprisingly, for most conditions tested with P[4], OD^350^ value after shaking for 6 h were elevated (was above 0.01) even in the presence of 0.025% PS-80 suggesting some undesirable level of aggregation. However, when the same set of conditions were tested in the presence of 150 mM NaCl and 10% sucrose +0.025% PS-80, aggregation was significantly reduced in all the samples containing P[4] antigen (Fig. 6c). Owing to limited availability of P[6] antigen, only one condition (10 mM PBS pH 7.2) could be tested for the effect of salt and results were comparable to P[4] (see Fig. 6b). Overall, these results demonstrated that addition of NaCl to the formulation (containing PS-80) might be necessary for the bulk storage of these antigens to better mitigate agitation-induced aggregation.

**Figure 7:**
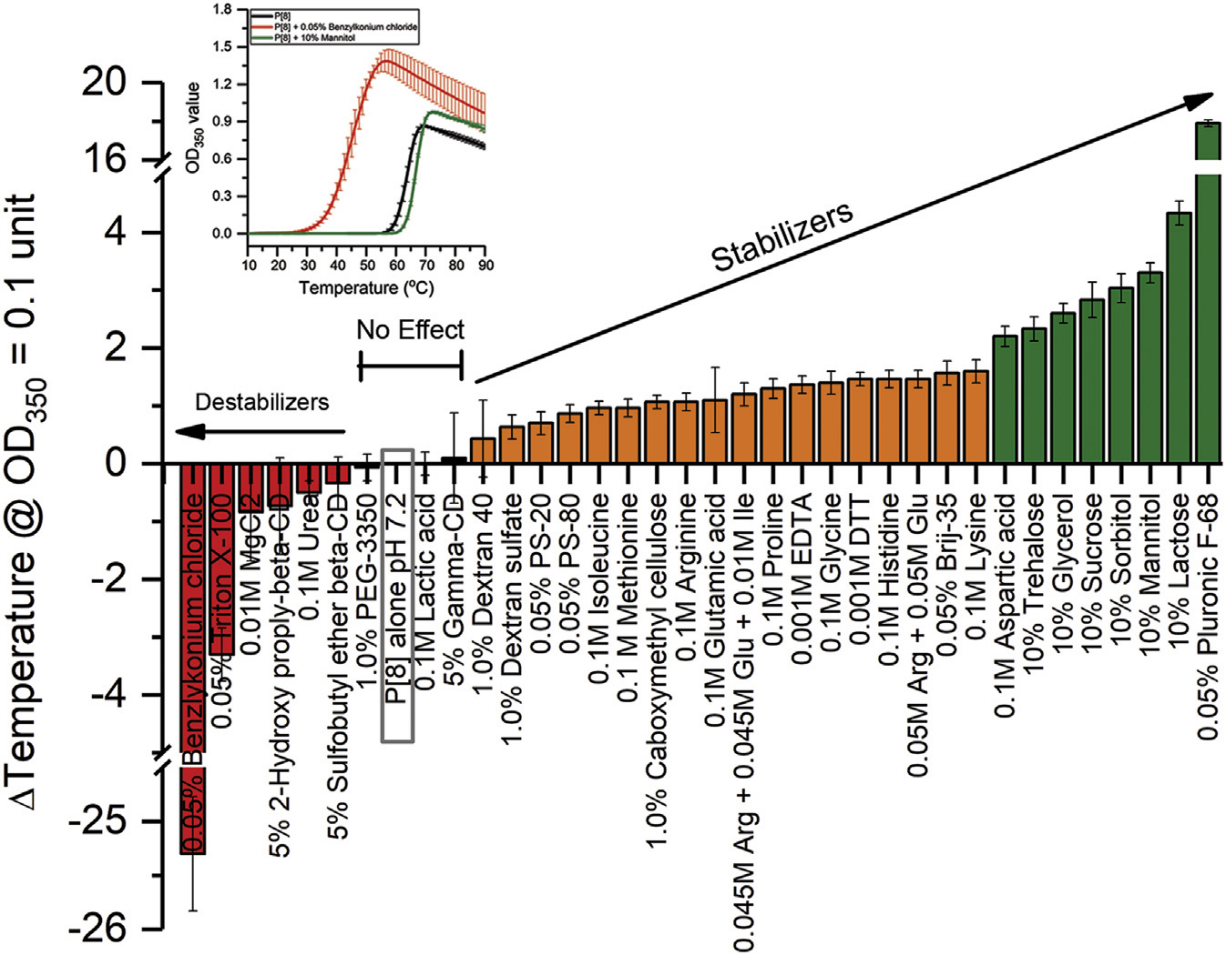
Excipient screening against thermal stress of P[8] protein antigen. OD350 studies of 0.1 mg/mL P[8] solution subjected to thermal stress from 10° C to 90° C in base buffer (10 mM PBS pH 7.2) and in base buffer containing different excipients. Average delta temperature (ΔT) value at which OD350 reaches 0.1 absorbance unit is shown and error bars represent 1SD from triplicate experiments. Excipients are rank ordered from lowest to highest ΔT value suggesting lowest to highest stability. Excipients in green, orange, gray, and red resulted in large increase, moderate increase, no effect, and decrease in stability, respectively. The inset shows OD350 versus temperature plots of a representative stabilizing @@@ (green line) and destabilizing (red line) excipient as compared to P[8] alone (black line) in base buffer.

Owing to the susceptibility of NRRV antigens toward aggregation under thermal stress (see companion paper by Agarwal et al.),^22^ we also screened the same set of 35 excipients with P[8] for their ability to minimize thermally induced aggregation. Figure 7 inset shows representative OD^350^ versus temperature plot for P[8] antigen alone and in the presence of a stabilizing and destabilizing excipient. The T^onset^ value for aggregation was defined as the temperature to reach OD^350^ value of 0.1, and ΔT@ OD^350^ of 0.1 value was defined as the difference between T^onset^ value for P[8] antigen with versus without excipient (thus, a positive ΔT shows a stabilizing effect of the excipient and a negative ΔT shows destabilization). As expected, carbohydrates and polyols showed stabilizing effect against thermal stress, and Pluronic F-68 showed dramatic stabilization of the P[8] antigen (Fig. 7). Similar to shaking stress, a subset of most stabilizing excipients were tested with P[4] and P[6] (data not shown). Based on these results, sucrose, trehalose, mannitol, sorbitol, and Pluronic F-68 were selected as initial “hits” to stabilize P[8] antigen under thermal stress. Sucrose was preferred over trehalose, mannitol, and sorbitol (even though all 3 showed similar or slightly better thermal stability profiles compared to sucrose) because of cost or the known tendency of these additives to crystallize out during freezing and thawing.^31-33^ In addition, equal amounts (% w/v) of the 2 polyols (vs. sucrose or trehalose) impart higher solution osmolality which is less desirable for parenteral administration due to their hypertonic nature.^34,35^

### Evaluation of Candidate Formulations for Bulk Drug Substance of Three NRRV Antigens

Eight candidate formulations (F2-F9) listed in Table 2 were designed based on the aforementioned results. Phosphate and HEPES buffer systems were chosen because of their trend as better stabilizing agents compared to histidine against agitation stress (Fig. 5c). Lower concentrations of phosphate were selected due to its known incompatibility with the aluminum-based adjuvant, Alhydrogel, which will be added as part of the drug product formulation (Agarwal et al., manuscript under review). No apparent effect of pH was observed in the agitation studies (Fig. 5c) and pH 7.2 was selected due to close proximity to physiological condition for compatibility with parenteral injection. Salt (NaCl) was selected as tonicifying agent based on its synergistic effect with PS-80 in mitigating shaking-induced aggregation of P[4] (Fig. 6c). Sucrose was also chosen because it enhanced the thermal stability of each NRRV antigen (Fig. 7) and could act as cryoprotectant during FT stress. As shown in Table 2, the osmolality values for each formulation were within an acceptable range for parenteral administration.

**Table 2:** Composition and Osmolality Values of 8 Candidate Frozen Liquid Formulations for Each NRRV Antigen for Their Individual Bulk Storage

F #	Formulation Components (pH 7.2)	Osmolality (mOsm)
1	1 mM sodium phosphate +150 mM NaCl (current bulk formulation)	270 ± 1
2	1 mM sodium phosphate +150 mM NaCl +0.025% PS-80	268 ± 1
3	1 mM sodium phosphate +10% w/v sucrose +0.025% PS-80	328 ± 2
4	5 mM sodium phosphate +150 mM NaCl +0.025% PS-80	297 ± 4
5	5 mM sodium phosphate +10% w/v sucrose +0.025% PS-80	330 ± 8
6	1 mM sodium phosphate +7.5% w/v sucrose +50 mM NaCl +0.025% PS-80	343 ± 4
7	5 mM sodium phosphate +7.5% w/v sucrose +50 mM NaCl +0.025% PS-80	308 ± 4
8	5 mM HEPES +10% w/v sucrose +0.025% PS-80	308 ± 4
9	5 mM HEPES +7.5% w/v sucrose +50 mM NaCl +0.025% PS-80	331 ± 2

Osmolality values are from triplicate measurements and error bars represent 1 SD.

**Figure 8:**
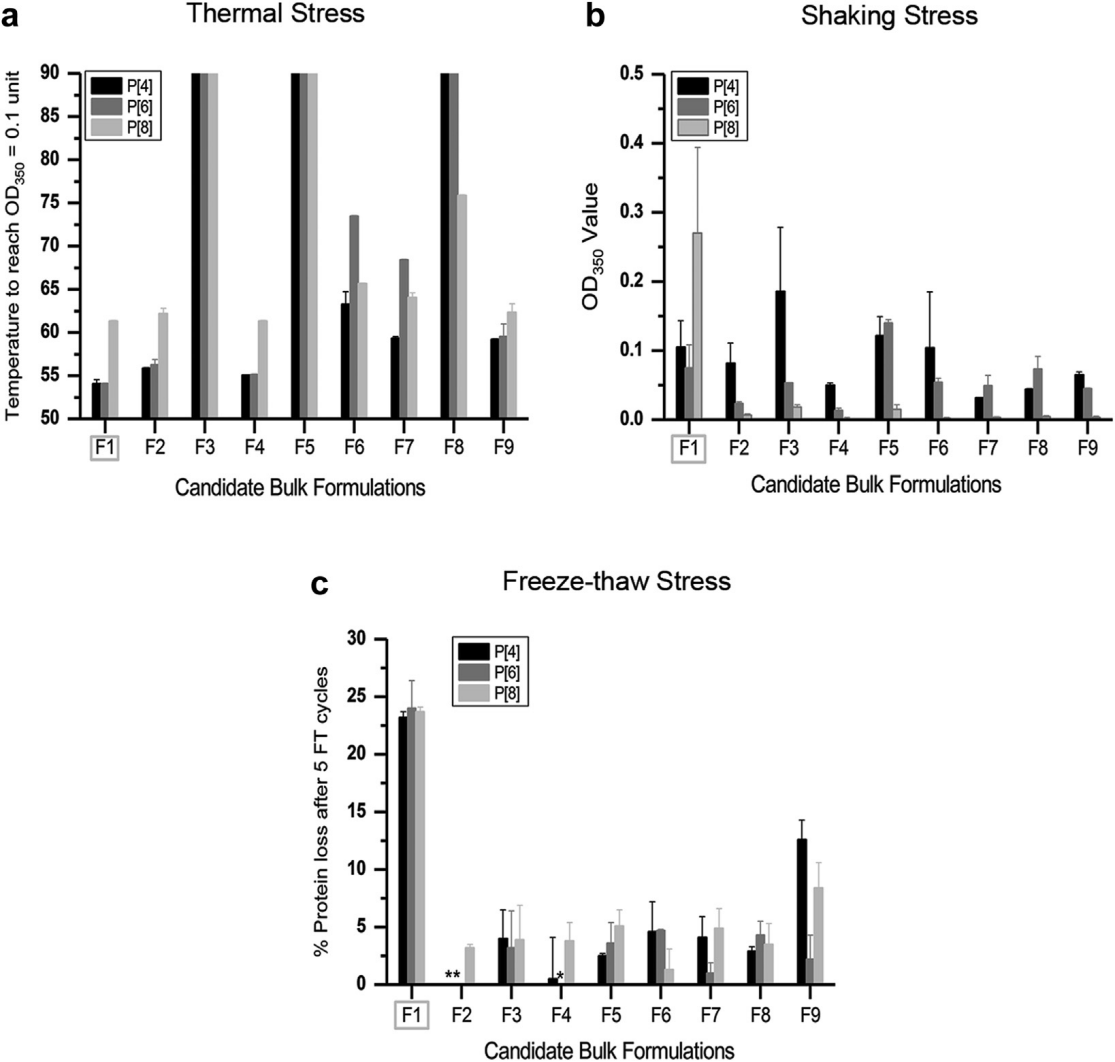
Comparison of candidate bulk formulations (F2-F9) versus current bulk formulation (F1), of individual NRRV antigens against (a) thermal stress, (b) shaking stress, and (c) freeze-thaw stress as measured by UV-visible spectroscopy analysis. Error bars represent 1 SD from triplicate experiments. Refer to Table 2 for the composition and osmolality of each candidate formulation. *No protein loss was observed for P[6], **no protein loss was oberved for P[4] and P[6].

**Figure 9:**
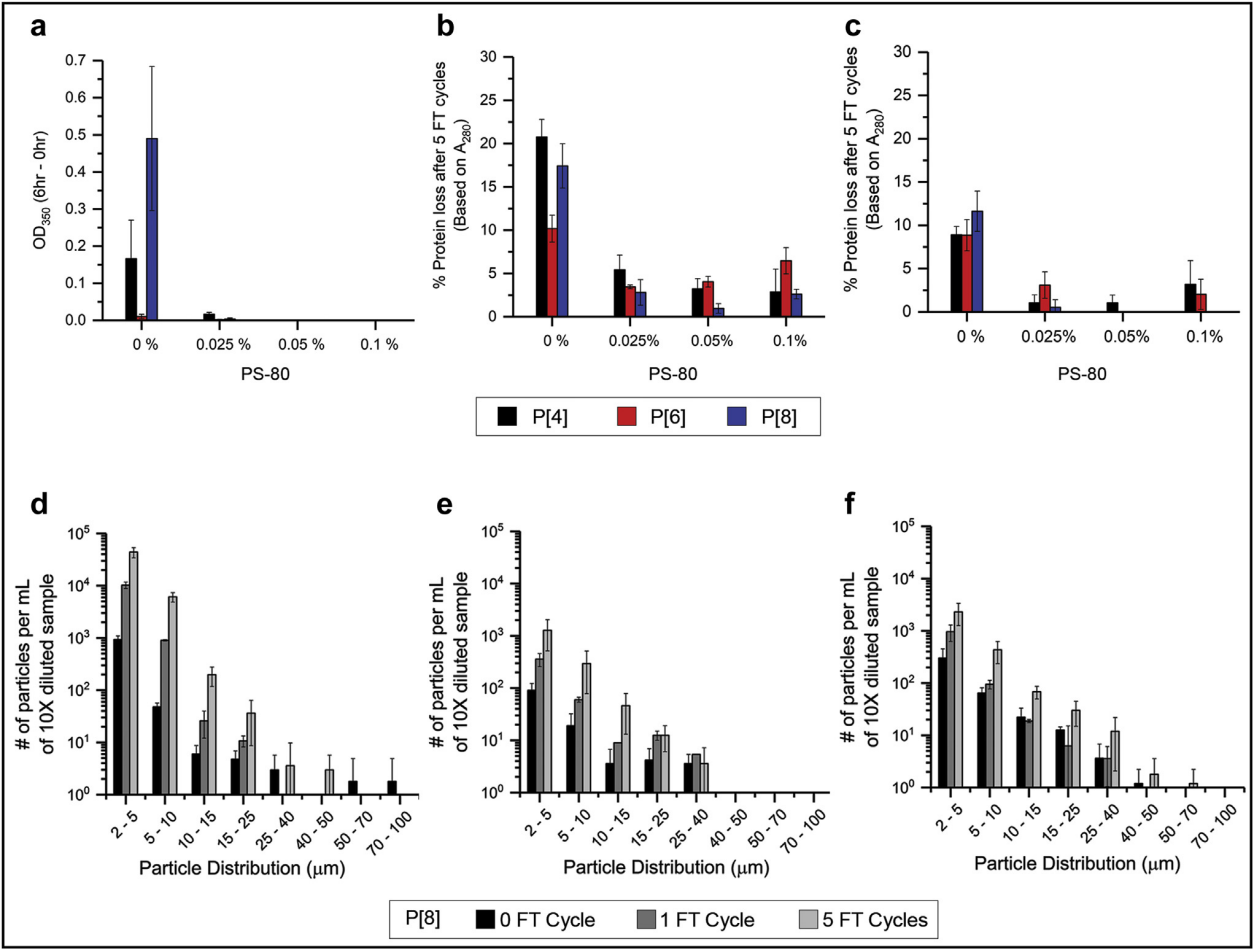
Effects of PS-80 concentration during freeze-thaw and shaking stresses on physical stability of NRRV antigens in current bulk formulation. (a) OD350 value after shaking each antigen for 6 h at 0.15 mg/mL protein concentration. Percent protein loss after 5 FT cycles at (b) 0.15 mg/mL and (c) 0.4 mg/mL protein concentration. Subvisible particle distribution analysis of P[8] antigen as measured by MFI after 0, 1, and 5 FT cycles in (d) current (1 mM sodium phosphate, 150 mM NaCl, pH 7.2) bulk formulation and 2 candidate bulk formulations, (e) 1 mM sodium phosphate, 150 mM NaCl, 0.05% PS80, pH 7.2, and (f) 10 mM histidine, 150 mM NaCl, 0.05% PS80, pH 6.8. Error bars represent 1 SD from triplicate experiments.

The candidate formulations (F2-F9) were then tested along with the current formulation (F1) for their ability to mitigate aggregation due to thermal, shaking, and FT stresses as shown in Figure 8. Each candidate formulation containing sucrose showed better thermal stability as compared to the current formulation F1 (Fig. 8a). For shaking stress study, 0.025% PS80 was able to reduce particle and aggregate formation for P[8] antigen but was not so effective for the other 2 antigens and warranted further examination (Fig. 8b). Addition of PS-80 to the candidate formulations was able to miti-gate protein loss for each antigen due to FT stress (albeit to a lesser extent with F9) as shown in Figure 8c. Different PS-80 concentrations (0.025%, 0.05%, and 0.1%) were examined at 0.15 mg/mL protein concentrations for each NRRV antigen to optimize the final PS-80 amount under shaking and FT stresses. As shown in Figure 9a (shaking stress), addition of 0.025% PS-80 was able to significantly reduce aggregation for P[4] and P[8] antigens (P[6] antigen was already least prone to aggregation). No notable increase in OD^350^ was observed with 0.05 or 0.1% PS-80 for each NRRV antigen. FT stress of 5 cycles caused low levels but measurable protein loss for each antigen at 0.15 mg/mL even in the presence of 0.1% PS-80 in the formulation as shown in Figure 9b. Because part of this loss could potentially be attributed to protein adsorption to the plastic tubes used in the study, we repeated the FT study at a higher protein concentration (0.4 mg/mL). A notable reduction in the percentage protein loss was observed at 0.4 mg/mL in the control (without PS-80) as well as PS-80—econtaining samples (Fig. 9c). For example, 0.05% PS-80 was most effective in mitigating protein loss for P[6] and P[8] antigens, and minimized loss for P[4] antigen. Thus, 0.05% PS-80 was added to candidate drug substance formulations.

The next goal was to test the selected candidate formulations to minimize or mitigate particle formation during FT stress under conditions which are more likely to occur during manufacturing and storage of the bulk drug substance materials. Figures 9d-9f shows subvisible particle distribution for P[8] antigen after 0, 1, or 5 FT cycle(s) in the current bulk formulation (Fig. 9d) and 2 candidate formulations (Fig. 9e—current formulation +0.05% PS-80, Figs. 9f—10 mM Histidine 150 mM NaCl 0.05% PS-80 pH 6.8). Most of the subvisible particles formed were in the size range 2-5μm and as expected the number of particles increased from 0 to 5 FT cycle. The 2 candidate formulations containing PS-80 helped in reducing subvisible particle formation by about 10- to 30-fold. Similar observations were made for P[4] and P[6] antigens and least number of subvisible particles were observed for P[4] out of the 3 antigen in the current formulation (Sup. Fig. S2). Visual assessment of FT stressed and control P[8] samples in the current and candidate formulations revealed no visible particle formation in these small scale experiments (data not shown). Finally, minimal to no changes were observed between the 3 formulations of each NRRV antigen upon FT stress with regard to mass loss, soluble aggregate distribution (from SEC analysis), overall conformational stability, or chemical modifications (data not shown).

## Discussion

The major goal of this work was to characterize and mitigate aggregation and particle formation of the 3 NRRV protein antigens during handling and storage as bulk drug substance (before formulation and fill-finish to manufacture the final vaccine drug product in vials). Formation of visible particles leading to precipitation was a concern associated with these antigens during early process development, especially during handling and FT in larger volumes (data not shown). It is important to note that because these antigens are already in clinical trials, a major constraint during this study was to minimize the change to current formulation to ensure the NRRV program’s progress is not hindered while ensuring optimal protein stability. Aggregates are widely studied as product-related impurities in biopharmaceutical drug candidates because they can be associated with potential immunogenic characteristics known to reduce the efficacy, for example, by generating anti-drug antibody responses.^36-39^ In the case of vaccine bulk protein antigens, large aggregates and particles can lead to protein loss during process development (e.g., during filtration) thus affecting the productivity and cost of vaccine production.

### Colloidal Stability Comparison and Aggregate/Particle Characterization

Although the P[8] antigen showed highest conformational stability compared to P[4] and P[6] (see companion paper, Agarwal et al.), it is also the most susceptible of the 3 antigens to shaking-or agitation-induced aggregation. This is likely due to lower colloidal or interfacial stability, or both of the P[8] antigen. It is possible that the thermal stress versus shaking stress (i.e., exposure to air-liquid interface, bubble entrapment, etc.) generate different types of partially unfolded protein states leading to different aggregation propensities. For example, such species generated under shaking stress could potentially have higher levels of exposed hydrophobic residues or regions with a greater tendency to interact and form multimers. It was interesting to compare the higher colloidal stability of P[4] and P[6] versus the P[8] antigen, although they share 66%-80% sequence homology. This result highlights the potential use of point mutations and protein engineering to improve pharmaceutical properties and developability of candidates without compromising their biological activity.^40^ Comparative second virial coefficient (B^22^) measurements can be made in the future, when sufficient material is available of each antigen, which would potentially be a good qualitative indicator of the differences in colloidal stability of these protein antigens.^41,42^ With limited material, however, we were able to compare the relative solubility ranking of the 3 antigens using AS^midpt^ values from the AS precipitation assay. The PEG precipitation assay (using a macromo-lecular crowding agent polyethylene glycol), which is widely used to screen monoclonal antibody candidates to assess their relative solubility under different formulation conditions,^43,44^ did not lead to notable precipitation with these 3 protein antigens (likely due to interaction between the protein and PEG, data not shown). We observed salting-out of each antigen in similar range of AS and interestingly lowest AS^midpt^ was observed for P[6] which is also most hydrophobic among the 3 antigens (see companion paper, Agarwal et al.^22^). Similar AS^midpt^ values were observed for P[4] and P[8] antigens. The relative rank ordering of solubility obtained from AS precipitation assay reflects the propensity of native state of a protein to self-associate. Thus, for these 3 protein antigens, the aggregate and particle formation pathway (during FT, thermal or shaking-induced stresses) is likely governed by the nature and extent of formation of structurally altered or partially unfolded protein states of the protein and not by protein-protein interactions in the native state.^45^

To this end, we then characterized the nature and composition of the NRRV protein within aggregates and particles. We utilized a limited shake stress test to evaluate the aggregates and particles when they just started to form, but most of the protein was still in native-like state. This enabled us to capture the initial formation of protein aggregates and particles for each antigen. The results showed overall similar characteristics for the 3 antigens in isolated aggregates or particles. For example, most aggregates were fibrillar in morphology with opaque nature, showed increased levels intermolecular β-sheet content, and some loss of native secondary structure content was recorded. Increased exposure of apolar regions was observed by ANS fluorescence studies and formation of aggregates containing non-native disulfide bonds (which were reducible in nature) were seen by SDS-PAGE analysis. Interestingly, the physicochemical characteristics of protein within the isolated aggregate or particle were similar in nature to the protein within aggregates generated for an IgG under different stress conditions as reported previously in our laboratories.^28^ These results further support the aggregation pathway of the NRRV antigens proceeds via formation of structurally altered protein intermediates (as described previously by a combination of FTIR, ANS fluorescence, and SDS-PAGE analyses).

### Excipient Screening, Optimization, and Formulation Development for Bulk Drug Substance Storage

During excipient screening and optimization studies with P[8], 0.025% PS-80 or Pluronic F-68 were effective in preventing shaking-induced aggregation either alone or in combination with sucrose and no NaCl containing formulations (Fig. 5c). Mechanisms by which nonionic surfactants (such as PS-80 and Pluronic F-68) and carbohydrates (such as sucrose) stabilize proteins against different stresses are well documented in the literature.^46,47^ Nonionic surfactants such as PS-80 and Pluronic F-68 are known to out-compete protein molecules for air-liquid, liquid-solid interfaces thus pre-venting protein structural alterations due to surface adsorption leading to non-native aggregate formation.^46^ Sucrose, on the other hand, is well-known to stabilize proteins by a preferential exclusion mechanism; the sugar molecules increase the free energy of the unfolded state (as compared to the native state of the protein), and thus, the native state of the protein is favored.^47^ Owing to limited material availability, only a subset of the stabilizing excipients identified with P[8] were tested with P[4] and P[6]. This challenge is not uncommon during early formulation development when only a few milligrams of material are available. Because P[8] was available in larger quantities, and because the 3 bulk antigens will be coformulated with aluminum adjuvant as a trivalent vaccine drug product, it was a reasonable and efficient approach to avoid screening each excipient with each of the 3 antigens.

Eight candidate bulk formulations (Table 2) were identified for the NRRV antigens ranging from minimal changes (such as addition of PS-80 to the current formulation, F1) to more major changes (such as changing the buffering and tonicifying agents). Each of these new formulations improved NRRV antigen stability against shaking, FT, and thermal stresses (Fig. 8). We further optimized the concentration of PS-80 and found 0.05% to be optimum concentration for each NRRV antigen to minimize shaking-induced aggregation and protein loss due to FT stress (Fig. 9). Notable levels of aggregation were observed for P[4] and P[6] antigens after shaking stress as well as significant protein loss was observed for each of these 2 NRRV antigens after 5 FT cycles (Fig. 8). It is important to note that the antigens were subjected to accelerated conditions (5 FT cycles) versus what they would normally encounter (1-2 FT cycles) during routine preparation. In addition, some protein loss during FT can be attributed to protein adsorption to plastic tubes used in the study (and % loss appears higher since the study was conducted at only 0.15 mg/mL protein concentration to conserve material). Lower percentage levels of protein loss were observed at higher protein antigen concentrations (>0.4 mg/mL) that will be targeted for future batches of the protein antigens.

Owing to limited real-time stability data with liquid formulations of protein-based bulk drug substances during early development, such bulks are often kept frozen (—80° C) during long-term storage to avoid degradation by reducing the mobility of molecules and mitigating transportation stress (shaking or agitation stress), for example, between the vaccine bulk and drug product manufacturing sites. Thus, stabilization of the 3 antigens against FT stress is critical because such treatments expose proteins to ice-water interfaces, cryoconcentrations (concentration gradients of protein and excipients across the container), pH shifts, and temperature fluctuations, which could lead to both physical and chemical degradation of the protein antigens.^48-51^ Addition of 0.05% PS-80 to the current formulation was able to mitigate subvisible particle formation till 5 FT cycles for each antigen (Fig. 9c). Formation of visible particles and precipitation during handling and on thawing of frozen NRRV antigen bulk solutions was observed in larger volumes in a suboptimal buffering system (data not shown). This was the major concern with the current formulation for bulk storage of these NRRV antigens because bulk drug substance typically undergoes mechanical agitation and at least one FT cycle (if stored frozen) before formulation steps (e.g., dilution, mixing with other antigens and adsorption to aluminum adjuvant to prepare the trivalent vaccine) and subsequent fill-finish into vials to produce a final vaccine drug product. No visible particles were observed for P[8] antigen in our scaled-down FT study in glass vials which might not be the most accurate representation of FT process in larger volume containers where freezing and thawing rates, surface-area to volume ratio, container type, head space, etc. can be substantially different.^52-54^

### Ongoing and Future Work

It is important to note that the candidate bulk formulations were designed so that minimal changes are required to the current formulation of the clinical NRRV vaccine (final drug product). Studies presented in this work were aimed not only at developing a better understanding the aggregation propensity of these 3 recombinant fusion-protein antigens, but also to identifying candidate formulations for bulk storage of the NRRV drug substance with improved physical stability (i.e., monovalent antigens). Based on the findings of this work demonstrating the susceptibility of the NRRV antigens to aggregation and particle formation under various stresses, combined with results from the companion paper showing Asn deamidation, Met oxidation, and non-native disulfide formation via a single Cys residue,^22^ the long-term storage of the NRRV bulk drug substances was recommended as a frozen liquid.

The formulated final vaccine drug product will be a trivalent vaccine drug product containing an adjuvant (Alhydrogel®) to enhance the immune response and potentially a preservative to enable multidose presentations. Ongoing work in our laboratories are therefore focusing on assessment of 2° C-8° C liquid formulations including interaction of the NRRV antigens with adjuvant, physicochemical, and immunochemical stability profiles of the antigens bound to adjuvant, and compatibility with antimicrobial agents (manuscript submitted). Thus, a key additional consideration for final selection of the frozen liquid bulk drug substance formulation during development will be compatibility with the vaccine drug product, both in terms of manufacturing and long-term stability of the trivalent, aluminum-adjuvanted vaccine candidate at various antigen doses.
