# Supplementary material for: Characterizing and Minimizing Aggregation and Particle Formation of Three Recombinant Fusion-Protein Bulk Antigens for Use in a Candidate Trivalent Rotavirus Vaccine
Source: J Pharm Sci. 2020 Jan 1;109(1):1–13. doi: 10.1016/j.xphs.2019.08.001 (PMC6941221; doi:10.1016/j.xphs.2019.08.001)
Supplement: Supplementary file 1 [file JXPHS-2019-j.xphs.2019.08.001-S1.docx]

**Supplemental Information**

**Characterizing and minimizing aggregation and particle formation of three recombinant fusion-protein bulk antigens for use in a candidate trivalent rotavirus vaccine**

Sanjeev Agarwal^a^, Neha Sahni^a^, John M. Hickey^a^, George A. Robertson^b^, Robert Sitrin^b^, Stanley Cryz^b^, Sangeeta B. Joshi^a^, and David B. Volkin^a,*^

^a^ Department of Pharmaceutical Chemistry, Vaccine Analytics and Formulation Center, University of Kansas, 2030 Becker Drive, Lawrence, Kansas 66047, USA

^b^ The Center for Vaccine Innovation and Access, PATH, 455 Massachusetts Ave NW Suite 1000, Washington, DC 20001, USA

*** Correspondence to:** David B. Volkin: 2030 Becker Drive, Lawrence, KS 66047, Email: volkin@ku.edu; Phone: 785-864-6262; Fax: 785-864-5736

**Current addresses:**

Sanjeev Agarwal: Amgen, Thousand Oaks, CA 91320, USA

Neha Sahni: GSK Vaccines, Rockville, MD 20850, USA

George A. Robertson: Cambra Consulting, 4895 Prince William Parkway, Suite 101 Woodbridge, VA 22039, USA

**Supplemental Methods**

**Colloidal Stability Studies Using Agitation Stress**

Each of the three NRRV recombinant protein antigens was dialyzed overnight at 4ºC in 10 mM sodium phosphate, 150 mM NaCl, pH 7.2 buffer, which is referred as “base buffer” in the text hereafter. The buffer-exchanged proteins samples in base buffer were subjected to shaking stress in 2 ml Fiolax clear, Schott (Lebanon, PA) glass vials with rubber stoppers (West Pharmaceutical, PA). The vials were filled with 0.4 ml of 0.15 mg/ml protein sample and were shaken sideways at 250 RPM for 6 hr. at RT. Stressed samples (6 hr.) in triplicate for each antigen and control samples (unstressed, 0 hr.) were assessed for the presence of aggregate/particles by OD_350_ value and the total number of sub-visible particles were counted by Micro-flow imaging (MFI) as described below.

For characterization studies of aggregates and particles, the samples were generated in the base buffer for each of the three NRRV antigens similar to colloidal stressed stability study described above. The vials were filled with 0.6 ml of 0.2 mg/ml protein sample and were shaken sideways at 250 RPM for 90 min at RT. These parameters were optimized to attain similar level of degradation in each antigen. The generated aggregates and particles were analyzed for their size, appearance, secondary structure, tertiary structure, and chemical composition by various analytical tools mentioned below.

## Visual Appearance and Turbidity

Samples were visually assessed for visible particles under Adelphi Apollo II liquid viewer (Adelphi Co., UK). Turbidity measurements were performed in triplicate using a Hach 2100 AN Laboratory Turbidimeter. Instrument calibration was achieved using standards in the range of <0.1 to 2,000 Nephlometric Turbidity Unit (NTU), and the turbidity values of the samples were corrected for the turbidity of the empty tube and base buffer.

**UV-Visible spectroscopy**

The UV-Visible absorption spectra of the NRRV samples were recorded before and after centrifugation (13,000 X g for 5 min) from 190-1100 nm using a 0.5 s integration time and 1 cm path length quartz cuvettes using an HP-8453 photodiode array detector (Agilent Technologies, Santa Clara, CA) equipped with Deuterium (D2) and Tungsten (W) lamps. The Beer-Lambert law was used to calculate the protein concentration using the extinction coefficient of a 0.1% solution of each antigen. Light scattering correction was applied to all the collected absorbance spectra using the manufacturer’s data analysis software (Chemstation UV-Vis analysis software, Agilent Technologies). The optical density value at 350 nm (OD350) was recorded from the uncorrected spectra.

**Micro-Flow Imaging (MFI)**

Sub-visible particles (1-100 µm) were measured and quantified using DPA-4200 flow microscope (Protein Simple, Santa Clara, CA) system equipped with a 100 µm silane coated flow cell. The instrument was calibrated using 10 µm polystyrene particle standards (Thermo Scientific) prior to analysis. The samples were carefully drawn up in a low protein binding, filter-tip pipette (Neptune Scientific, San Diego, CA) and analyzed using a flow rate of 0.17 ml/min. The purge volume for each measurement was 0.2 ml and measurements were made at ambient temperature. Particle free water was used to optimize illumination prior to each measurement.

**Resonance Mass Measurement (RMM)**

Archimedes particle metrology system (Affinity Biosensors, Santa Barbara, CA), equipped with Hi-Q microsensor was used to assess the total number and distribution of sub-micron particles (200 nm to 1 µm) in the samples. Prior to each sample run, clean baseline was obtained by cleaning the flow cell and sensor with 20% Contrad 20 and then flushing with particle free water. The accuracy of the sensor was determined by analysis of 1 µm polystyrene beads (Thermo-Scientific). Particle density of 1.37 g/ml was used, limit of detection was set to 0.03 Hz and measurements were made at ambient temperature. Samples were run for either 10 min or till the total particle count reached 300.

**Sedimentation Velocity Analytical Ultracentrifugation (SV-AUC)**

SV-AUC experiments were performed on the unstressed and stressed samples as per the method described earlier (see companion paper, Agarwal et al., 2019).^1^

**Size Exclusion Chromatography (SEC)**

Stressed and unstressed samples were subjected to SEC analysis as per the method described earlier (see companion paper, Agarwal et al., 2019).^1^

**Fourier Transform Infrared Spectroscopy (FTIR)**

FTIR spectra were collected for each of the stressed and unstressed sample as per the previous method (see companion paper, Agarwal et al., 2019).^1^ Qualitative comparisons (number or peaks and their position) were made between the in solution protein FTIR spectra vs the FTIR spectra of filtered particles from same sample obtained using FTIR microscopy technique (described below).

**Fourier Transform Infrared Microscopy**

The samples were filtered using 3 μm gold filters (Pall Corporation) which were pre-equilibrated by washing with 0.1 M NaOH. After filtration, samples were washed with ultrapure water, and dried overnight. A Bruker Hyperion FTIR Microscope with a 15X objective was used to image individual particles. Two-hundred-fifty-six scans were recorded from 600-4000 cm-1 with a viewing area of about 100 μm × 100 μm. OPUS (V6.5) software was used for baseline and atmospheric correction. The second derivative spectra were obtained using OPUS software and applying a nine-point Savitzky-Golay smoothing function.

**Extrinsic (ANS) Fluorescence Spectroscopy**

Samples were centrifuged at 13,000g for 5 min to separate the soluble and insoluble fractions. The pellet was re-suspended in base buffer. The final protein concentration in each of these supernatant and pellet components was 0.15 mg/ml. 8-Anilino-1-naphthalene sulfonate (ANS) was used as an extrinsic fluorescence probe with the A PTI QM-1 spectrofluorometer (Brunswick, NJ) equipped with a turreted four-position Peltier-controlled cell holder and a xenon lamp. The stock solution of ANS (1-Anilinonaphthalene-8-sulfonate) dye purchased from Sigma, Inc. (St. Louis, MO) was made at 25 mM in DMSO. Finally, a ratio of 25:1 (dye:protein) was maintained in the protein samples during fluorescence measurements. An excitation wavelength of 372 nm was used and emission spectra was collected from 400-600 nm at a 1 nm/s collection rate in 0.2 cm path length cuvette. Spectra for all the samples (supernatant and pellet of stressed and unstressed samples) of a particular antigen were collected by keeping the light intensity constant and this constant intensity was set using the supernatant of the unstressed sample at RT. The obtained emission spectra were corrected for the buffer blank and data were plotted using Origin (v 7.0) software.

**SDS-PAGE**

Prior to SDS-PAGE analysis, samples were centrifuged for 5 min at 13,000 X g, and the resulting supernatant and pellet were separated. Approximately, 2.5 µg of protein (supernatant or pellet) (+/-shaking) was mixed with 4X NuPAGE LDS sample buffer (Life Technologies). Nonreduced samples were incubated in dark for 10 min with 20 mM iodoacetamide and then boiled at 95°C for 10 min. For reducing conditions, 10 mM dithiothreitol (DTT) was added followed by incubation at 37ºC for 10 min. The samples were then separated using NuPAGE 12% Bis-Tris gel (Life Technologies) and MES running buffer by running at 150 V for 60 min. Staining of protein bands was done using Coomassie blue R250 (Teknova, Hollister, CA), followed by destaining with a mixture of 40% methanol, 10% acetic acid, and 50% ultrapure water. Gels were digitized using an Alphaimager (Protein Simple, Santa Clara, CA) gel imaging system.

**Ammonium Sulfate (AS) Precipitation Assay**

AS precipitation assay was performed for each antigen in base buffer at RT. Stock solution of 3.5 M AS was prepared in the same buffer and pH was adjusted to 7.2. Samples were prepared in a 96-well plate to a final protein concentration of 0.18 mg/mL by mixing appropriate amounts of protein stock solution, AS stock solution and buffer to a final volume of 125 µL. Samples were mixed and incubated at RT for 10 min before filtering through a 0.2 µm polystyrene filter plate (Corning #3504; Corning Life Sciences, NY) by centrifugation at 3000 RPM for 10 min. Thereafter, 80 µL of the filtrate was transferred to a 384-well UV Star microplate (Greiner #781801; Greiner Bio-one, NC) and absorbance was measured at 280 nm on a SpectraMax M5 UV-Visible plate reader. Protein concentration (mg/mL) versus AS molar concentration (M) data were fit to a Boltzmann sigmoidal curve function as described by Yamniuk et al using Origin 2017 to obtain AS mid-point value (AS_midpt_) for each antigen.^2^

**Excipient Screening, Optimization, and Formulation Development Studies**

*Excipient screening studies using shaking stresses*- Frozen P[8] protein samples were thawed and buffer exchanged in base buffer. Stock solutions (2x) of 35 pharmaceutical excipients were prepared in base buffer, and a stock solution of NaCl (salt) (10x) was also prepared which consisted of 10 mM sodium phosphate, 1.5 M NaCl, pH 7.2 buffer. For each sample for excipient screening, an excipient of interest, protein stock solution, salt stock solution, and base buffer were combined to achieve the desired concentration of each excipient. A final protein concentration of 0.15 mg/mL was used and samples were assayed in triplicate. Fiolax clear 2 mL, Schott (Lebanon, PA) glass vials were filled with 0.4 mL of 0.15 mg/mL protein and stoppered with sterile and coated stoppers (Part# 19700302, West Pharmaceutical, PA). The vials were then shaken sideways at 300 RPM for 6 hr. at room-temperature. Additionally, control vials were filled with 0.4 mL of each base buffer without protein and shaken under similar conditions. UV-Visible Spectroscopy and Micro-flow imaging (MFI) were used in excipient screening experiments as described above. The excipients that showed stabilizing effect on P[8] antigen were tested for their effect on P[4] and P[6] antigens. Not all of the excipients could be tested with P[4] and P[6] antigens due to limited material available for these antigens.

*Concentration optimization of lead excipients for their stabilizing effect:* concentration of the identified lead excipients– PS-80, 2-OH propyl β-CD, Pluronic F-68 (Figure 5A) was optimized using P[8] antigen under shaking stress using MFI assay.

*Salt and candidate excipient(s) combination optimization:* increasing concentrations of salt (NaCl) from 0 to 150 mM were screened with different combinations of lead excipients for their stabilizing effect on the P[8] antigen. Different combinations (C1 – C14) selected for the lead excipients at their optimized concentration are mentioned in table below Figure 5B and MFI assay was used to measure sub-visible particles of shake stressed samples.

*pH and buffer optimization studies:* selected combination of excipients (0.025% PS80 + 10% Sucrose) was further tested in different buffers (1 mM sodium phosphate, 10 mM sodium phosphate, 10 mM histidine, and 10 mM HEPES) at different pH conditions 6.5, 6.8, 7.2, and 7.5 to study their effect on P[8] under shaking stress. Based on the effect of these buffer conditions on P[8] antigen, a subset of conditions (1 mM sodium phosphate pH 6.5 and 7.2, 10 mM sodium phosphate pH 6.5 and 7.2, 10 mM HEPES pH 6.5 and 7.2, and 10 mM sodium phosphate with 150 mM NaCl pH 7.2) were down selected to test with P[4] and P[6] antigens.

*Excipient screening studies with P[8] under thermal stress:* thermal stability of the P[8] antigen was also tested in the presence of the 35 pharmaceutical excipients to assess their stabilizing or destabilizing effect. A protein concentration of 0.10 mg/mL was used and samples were stressed by OD_350_ assay as a function of temperature as described in the companion paper by Agarwal *et al.,* 2019.^1^

*Shaking, freeze-thaw (FT), and thermal stress studies of candidate bulk formulations:* based on the information gained from the above studies, 8 candidate bulk formulations were designed (Table 2) and tested for the stability of the three antigens under forced shaking, FT, and thermal stress conditions. Shaking and thermal stress studies were conducted as previously described here. For FT study, stress consisted of five cycles of freezing each sample at -80˚C and thawing at room-temperature. 200 µL of protein samples at 0.15 mg/mL in 1.5 mL Eppendorf tubes were used in this study and protein loss after five FT cycles was determined by measuring absorbance at 280 nm.

*Further optimization of PS80 concentration:* based on the protein loss data during FT study with 8 candidate bulk formulations, three different concentrations of PS80; 0.025%, 0.05% and 0.1% were further tested against FT stress (5 cycles) at protein concentration of 0.15 and 0.40 mg/mL for each of the three antigens in 1mM sodium phosphate 150 mM NaCl pH 7.2 buffer (current formulation buffer). We also evaluated these different PS80 concentrations (0.025%, 0.05% and 0.1%) against shaking stress for each antigen in current formulation buffer with the same method as described above.

*Additional FT studies with candidate bulk formulations for the three NRRV antigens:* each of the three NRRV antigens in the current formulation and two candidate formulations (1 mM sodium phosphate 150 mM NaCl 0.05% PS80 pH 7.2, 10 mM histidine 150 mM NaCl 0.05% PS80 pH 6.8) were subjected to 1 and 5 FT cycles in Eppendorf tubes at ~1 mg/mL concentration. Samples were subjected to visual inspection, SEC, UV-Visible spectroscopy, MFI, and DSC.

**Supplementary Figure S1.** (A, C) Total sub-visible particles, and (B, D) OD_350_ value of 0.15 mg/mL P[4] and P[6] solutions after shake stressed for 6 h. Studies were conducted in base buffer (black bar, highlighted in box) and in base buffer containing different excipients. Excipients are rank ordered from lowest to highest total sub-visible particles or OD_350_ value suggesting highest to lowest stability. Excipients in green, orange, and red resulted in large increase, no effect, and decrease in stability, respectively. Top row shows data for P[4] and bottom row for P[6] antigen, error bars represent 1 SD from triplicate measurements.


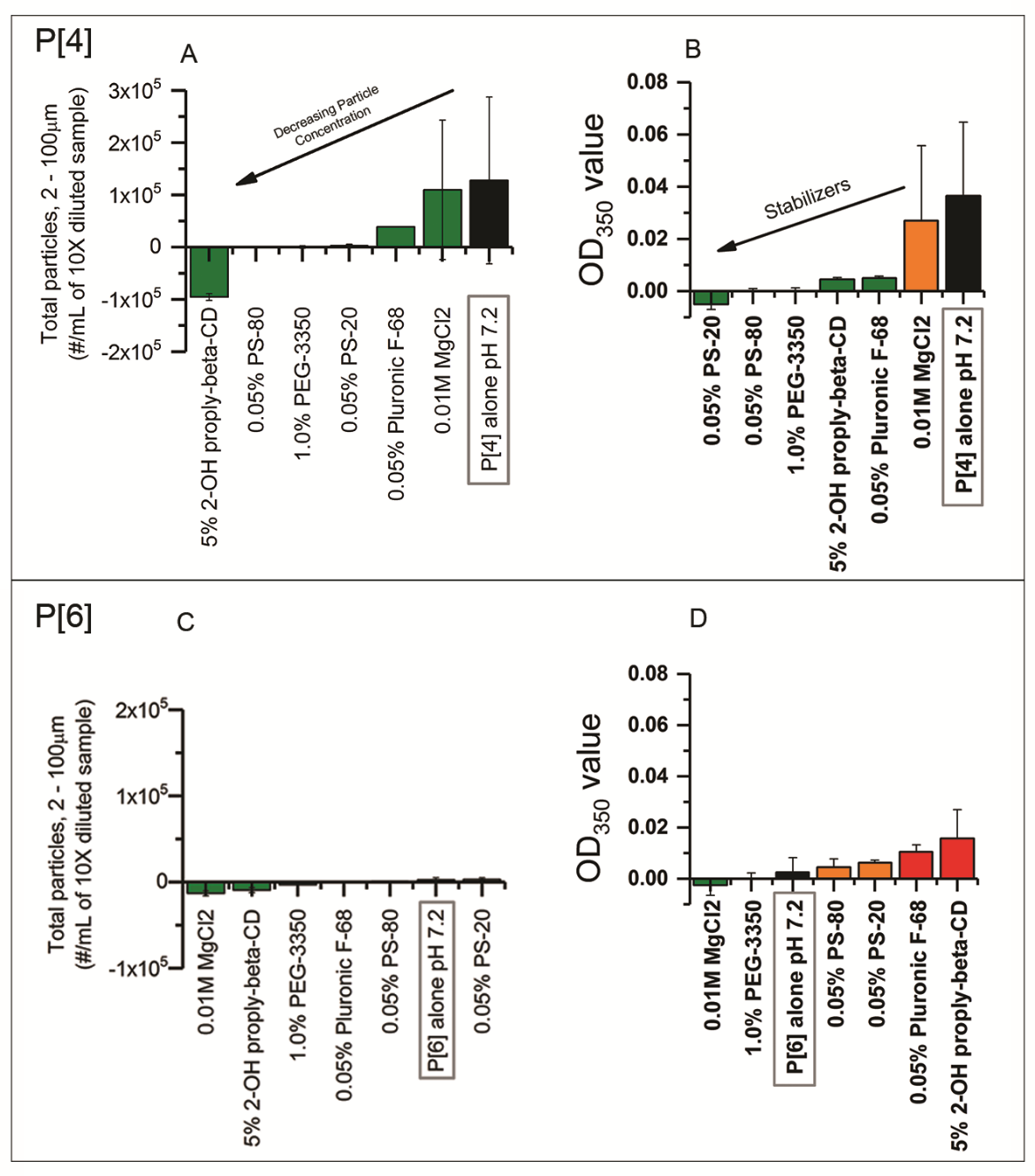


**Supplementary Figure S2.** Sub-visible particle distribution analysis of P[4] and P[6] antigens after 0, 1, and 5 FT cycles as measured by MFI. Samples in (A, D) current formulation (1mM sodium phosphate 150 mM NaCl pH 7.2) and two candidate formulations, (B, E) 1mM sodium phosphate 150 mM NaCl 0.05% PS80 pH 7.2, and (C, F) 10 mM histidine 150 mM NaCl 0.05% PS80 pH 6.8. Top row shows data for P[4] and bottom for P[6] antigen. Error bars represent 1 SD from triplicate experiments.


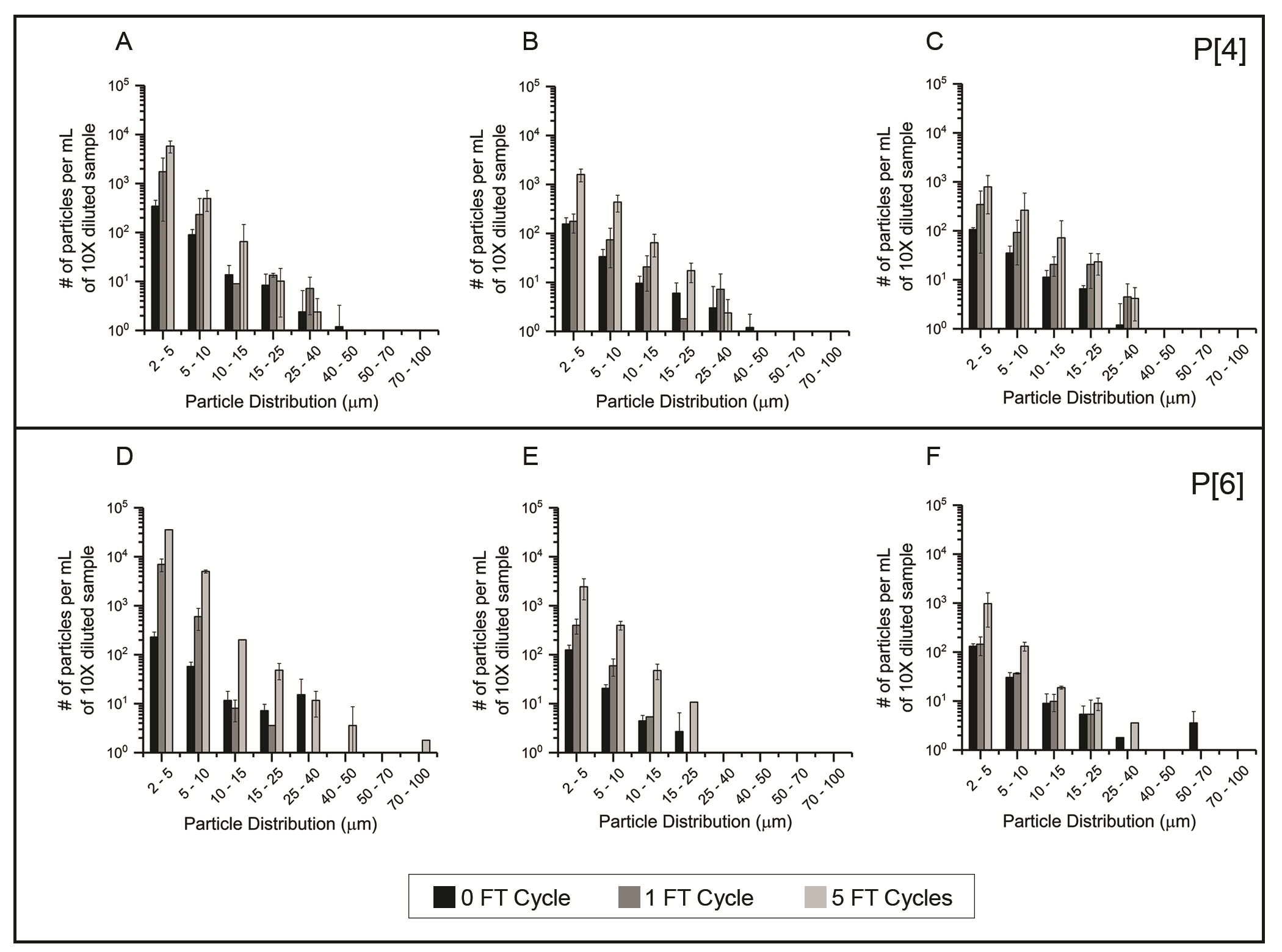


**References in Supplemental Information**

1. Agarwal S, Hickey JM, Sahni N, Toth RT, Robertson GA, Sitrin R, Cryz S, Joshi SB, Volkin DB 2019. Recombinant subunit rotavirus trivalent vaccine candidate: physicochemical comparisons and stability evaluations of three protein antigens. Journal of Pharmaceutical Sciences - In press.

2. Yamniuk AP, Ditto N, Patel M, Dai J, Sejwal P, Stetsko P, Doyle ML 2013. Application of a kosmotrope-based solubility assay to multiple protein therapeutic classes indicates broad use as a high-throughput screen for protein therapeutic aggregation propensity. J Pharm Sci 102(8):2424-2439.
